# A Comprehensive, Epidemiological and Ecological Descriptive Study on Vitamin D Status in Iran (308005 People, from 2009–2018)

**Published:** 2019-04

**Authors:** Dariush D. FARHUD, Atefeh MEHRABI, Abdolfattah SARAFNEJAD, Hamid Reza SADEGHIPOUR, Abbas RAHIMIFOROUSHANI, Mohammdad Bagher ROKNI, Keyvan MAJIDI, Ahad ALIZADEH, Marjan ZARIF-YEGANEH, Maryam JALALI, Mahmoud JALALI, Ali Akbar AMIR ZARGAR, Farideh KHOSRAVI, Amir MOMENI, Mohammad KHAZENI, Asadallah HENDIANI, Mehdi AHMADI, Alireza DEHSHIRI, Payam RASOOLI

**Affiliations:** 1. School of Public Health, Tehran University of Medical Sciences, Tehran, Iran; 2. Department of Basic Sciences/Ethics, Iranian Academy of Medical Sciences, Tehran, Iran; 3. Farhud Genetic Clinic, Tehran, Iran; 4. School of Advanced Medical Sciences, Islamic Azad University, Tehran Medical Branch, Tehran, Iran; 5. Noor Pathobiology Laboratory, Tehran, Iran; 6. Department of Immunology, School of Public Health, Tehran University of Medical Sciences, Tehran, Iran; 7. Department of Physiology, School of Medicine, Tehran University of Medical Sciences, Tehran, Iran; 8. Department of Epidemiology and Biostatistics, School of Public Health, Tehran University of Medical Sciences, Tehran, Iran; 9. Cellular and Molecular Endocrine Research Center, Research Institute for Endocrine Sciences, Shahid Beheshti University of Medical Sciences, Tehran, Iran; 10. Department of Biochemistry, School of Medicine, Tehran University of Medical Sciences, Tehran, Iran; 11. Booali Pathobiology Laboratory, Qom, Iran; 12. Department of Virology, School of Public Health, Tehran University of Medical Sciences, Tehran, Iran; 13. Payvand Teb va Narmafzar Company (PTN), Tehran, Iran

**Keywords:** Vitamin D deficiency, Epidemiology, Iran

## Abstract

**Background:**

Vitamin D is an essential substance for absorption of calcium and phosphorus from intestine so it is vital for muscles and skeletal development. Deficiency of this vitamin is pandemic. The vitamin D status depends on the different factors such as UV exposure, diet, and ecological features of living location, age and gender. The aim of this study was to describe the vitamin D level in different provinces of Iran and to investigate the association between vitamin D status and multiple variables.

**Methods:**

We collected the serum 25(OH)D (Vitamin D) level data of 308,005 people referred to different laboratories from 30 provinces of Iran and organized them by each province, year, age, gender, precipitation, latitude and longitude, and humidity over 10 yr (2009–2018). Data were analyzed to find out the correlation between age, gender, longitude and latitude, humidity and sum of precipitation.

**Results:**

West Azerbaijan had the highest level of vitamin D with a mean level of 33.24 and a standard deviation of 32.001, and North Khorasan had the lowest level with a mean level of 14.46 and a standard deviation of 8.980 among 30 provinces of Iran. The correlation between all studied variables (age, and gender, latitude and longitude, humidity, the sum of precipitation) was significant (*P*<0.001).

**Conclusion:**

The average total vitamin D level in Iran is 25.41 ng/ml, which is within the area of deficiency. Vitamin D is associated with age, and gender, latitude and longitude, humidity, the sum of precipitation. So changes in any of these variables can lead to vitamin D alteration.

## Introduction

Vitamin D (Calciferol) is a neurohormone that has important roles in development and strength of muscles and bones by its effect on calcium absorption from the gut (1, 2). It is mostly synthesized in the skin with sunlight UV radiation (UVB) exposure (3). A low amount of vitamin D is also provided by consumption of foods such as fish liver oil, egg yolk, and dairy products. So generally vitamin D level depends on a person’s lifestyle and the most important factor is the time of UVB exposure (4, 5).

Calciferol has a steroidal structure derived from cholesterol. Initially, in the skin, under the influence of the dehydrogenase enzyme, cholesterol becomes 7-dehydrocholesterol and then converts to cholecalciferol (vitamin D3) by ultraviolet radiation. The active form of vitamin D3 is 1, 25-dihydroxycholecalciferol. Activation of this vitamin occurs in the liver and kidneys. Enzymatically 25-hydroxycholecalciferol is produced by 25-hydroxylase (CYP2R1) in the liver and next it is transformed to 1, 25-dihydroxycholecalciferol (calcitriol) by 1α-hydroxylase (CYP27B1) in the kidney (6–9).

Vitamin D deficiency is a worldwide issue influencing people with every age and sex. Its deficiency is considered as 25(OH) D serum level <30 ng/ml. The duration of sunlight exposure, age, sex, diet, skin pigmentation, latitude, altitude, and air pollution are some factors affecting the vitamin D status (10, 11).

The prevalence of vitamin D deficiency varies in each geographical location based on different conditions, nearly 30% to 58.8% in different parts of the world (12). In North America non-Hispanic blacks has the lowest status of vitamin D while Hispanic whites have the highest. Besides, in Europe Nordic countries has better mean serum 25(OH) D than Mediterranean ones. In the Middle East despite abundant sunlight, vitamin D deficiency has a high frequency (13).

Vitamin D deficiency leads to problems related to bone and muscle, unexplained musculoskeletal pain, diabetes, autoimmune disorders such as multiple sclerosis, calcium-phosphorus metabolism disorders, cardiovascular diseases, cancer etc. (1, 9, 14–16). The daily requirement of vitamin D is about 400 to 800 UI. It may be necessary to use vitamin D supplements for prevention (17).

The aim of this study was to verify vitamin D status in 30 provinces of Iran with a glance to the age, gender, precipitation, latitude, and longitude and humidity classification of each one.

## Materials and Methods

### Location

**Fig. 1:**
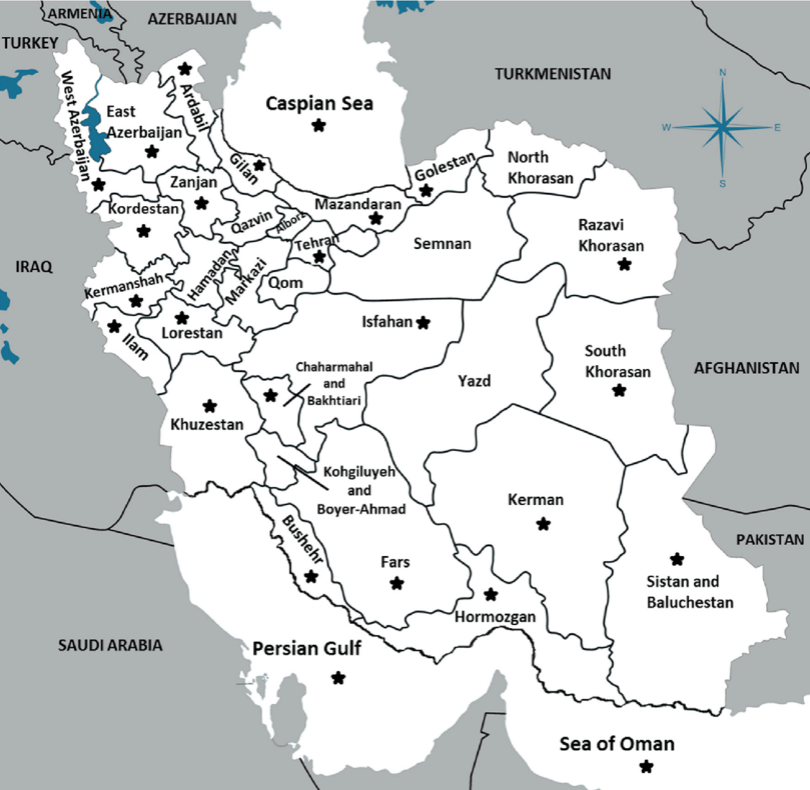
Location of provinces of Iran (20)

Iran with an area of 1,873,959 km^2^ and 31 provinces is considered a vast country (Fig. 1). It is located in Middle East (between 25N and 40N in latitude and between 44E and 64E in longitude) (18–20).

**Fig. 2:**
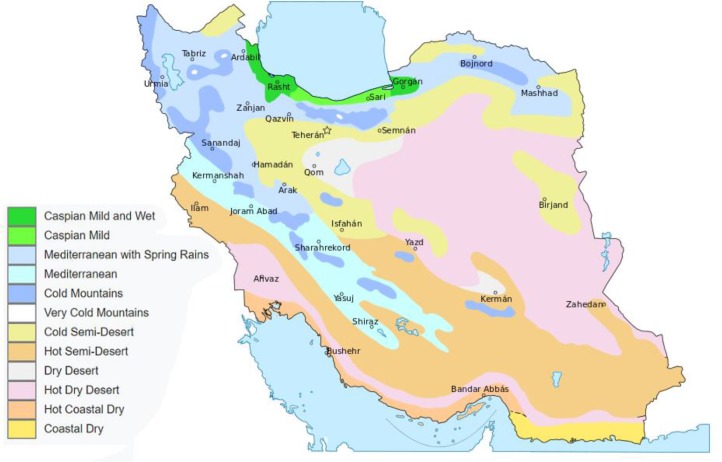
Ecological structure of Iran with capital of each province (24)

Iran has a diverse ecological structure, More than half of Iran is desert and semi-desert, and about one third is mountainous and a small part of Iran is composed of fertile plains (18). The climate of Iran mainly consists of four divisions including mild and humid (the South Coast of the Caspian Sea), cold mountainous (the northern slopes of the Alborz and the western slopes of Zagros), warm and dry (central Plateau), and warm and humid (southern shores) (Fig. 2) (18, 21). The temperature difference between the hottest and the coldest regions –in both summer and winter- is more than 50°C in the same day (22).

Iran is generally a dry land. The precipitation in Iran is very variable in different locations depend on latitude, longitude and other geographical features (23). The elevation is also very divers. The lowest is at the Caspian Sea (about −28 m) and the highest is Damavand peak (5,610 m) (24).

There are at least 10 major different ethnic groups (Table 1) in Iran with specific dialects, food habits, clothing and different lifestyles (Azaris, Kurds, Lors, Farses, Baluches, Turkmens, Arabs, Gilaks, Mazanis, and Afghans). Due to the variety of ethnicities and the effects of neighboring cultures, Iranian food is also very diverse.

**Table 1:** Different Ethnic groups of Iran and their location (25)

***Ethnicity***	***Original livings area***
Farses	Tehran, Khorasan, Isfahan, Shiraz, Kerman, Hamadan, Qom, Yazd
Azaris	West Azerbaijan, east Azerbaijan, Ardabil, Zanjan, Qazvin, Golestan
Kurds	Kurdistan, Kermanshah, Ilam, west Azerbaijan
Gilaks	Gilan, west of Mazandaran, Qazvin
Mazanis	Mazandaran, Golestan
Baluchis	Sistan and Baluchestan
Turkmens	Golestan, North Khorasan
Arabs	Khuzestan, Chaharmahal and Bakhtiari
Lors	Lorestan, Ilam
Afghans	Eastern area of Khorasan

Iran also has a variety of religious minorities, including Zoroastrian, Jews, Armenians, and Assyrians which live in different areas of Iran with their specific socioeconomic features (25).

### Sampling

In this descriptive study, we collected data from 308,005 samples in a period of 10 yr (2009 to 2018). We asked different laboratories from 31 provinces of Iran to send us their data in this timespan. At the end, data from 30 provinces were collected in excel files and were classified by year, province, age and gender. One province (Chaharmahal and Bakhtiari) did not cooperate in this project. Data about longitude and latitude, humidity and sum of precipitation were obtained from Iran Meteorological Organization (http://www.irimo.ir/eng/index.php).

The sampling was a passive one (retrospective) from patients referring to the diagnostic laboratories with different medical reasons, also checkups and by physicians with different specialties, all over the country.

### Statistical Analysis

All outliers were deleted and statistical analysis was performed only on remained 292,503 samples with SPSS Version 25 (Chicago, IL, USA) and R statistic software (Version 3.5.2). Data were expressed as means and standard deviation (SD). A value of *P*<0.05 was considered statistically significant.

There are different cutoff points for vitamin D status in publications and there is no constant range for it. We considered vitamin D amount of 30–100 ng/ml as sufficient, 10–29 ng/ml as deficient and vitamin D <10 ng/ml as severely deficient (26).

## Results

Vitamin D level of 308,005 people was collected from 30 provinces of Iran and after deleting outliers, the rest data (292,503 samples) were analyzed. We have measured the mean and standard deviation of the total group and each province separately.

The number of investigated samples in different provinces of Iran is represented in Table 2. The most portion of collected samples is first from Tehran with 64.1% followed by Qom with 23.8%. Other provinces are between 1.9% and 0.0% (Table 2).

**Table 2:** Distribution of collected samples in different provinces of Iran

***Province***	***Number of samples***	***Percent***
Alborz	5469	1.9
Ardabil	751	0.3
Bushehr	1854	0.6
Chaharmahal and Bakhtiari	0	0.0
East Azerbaijan	214	0.1
Fars	48	0.0
Gilan	668	0.2
Golestan	64	0.0
Hamadan	1380	0.5
Hormozgan	665	0.2
Ilam	510	0.2
Isfahan	1319	0.5
Kerman	518	0.2
Kermanshah	750	0.3
Khuzestan	3517	1.2
Kohgiluyeh and Buyer-Ahmad	10	0.0
Kordestan	3203	1.1
Lorestan	2966	1.0
Markazi	1116	0.4
Mazandaran	3963	1.4
North Khorasan	5	0.0
Qazvin	1717	0.6
Qom	69615	23.8
Razavi Khorasan	206	0.1
Semnan	1722	0.6
Sistan and Baluchestan	983	0.3
South Khorasan	48	0.0
Tehran	187629	64.1
West Azerbaijan	201	0.1
Yazd	824	0.3
Zanjan	568	0.2
Total	292503	100.0

The gender distribution of the studied sample in Iran in different years (2009 to 2018) is shown in Table 3. It is comprehensible that over the years, the frequency of women has been higher than men. Meanwhile, the trend in female number is decreased from 77.4% in 2009 to 68.1% in 2018. The percentage of male samples is increased from 22.6% in 2009 to 31.9% in 2018.

**Table 3:** Distribution of collected samples according to gender and year in Iran

***Year***	***Female***		***Male***	
	***Number***	***Percent***	***Number***	***Percent***
2009	8817	77.4	2573	22.6
2010	18256	77.5	5306	22.5
2011	23196	76.3	7193	23.7
2012	31585	74.8	10629	25.2
2013	29757	72.4	11322	27.6
2014	22242	72.0	8633	28.0
2015	24192	70.7	10013	29.3
2016	26926	67.7	12871	32.3
2017	21558	65.8	11196	34.2
2018	11294	68.1	5292	31.9
Total	217823	72.3	85028	27.7

The descriptive statistics including mean and standard deviation of vitamin D level, age, humidity and sum of precipitation during 2009–2018 in Iran is manifested in Table 4.

**Table 4:** Descriptive statistics of Vitamin D, age, humidity, and the sum of precipitation by year, in Iran

***Year***	***Vitamin D Level***		***Age (yr)***		***Humidity***		***Sum of precipitation***	
	***Mean***	***SD***	***Mean***	***SD***	***Mean***	***SD***	***Mean***	***SD***
2009	30.28	30.16	41.00	20.44	45.79	6.52	337.34	99.01
2010	28.10	27.94	40.78	20.36	43.76	7.12	346.52	95.72
2011	28.14	28.64	40.28	20.49	44.08	6.44	391.42	116.31
2012	27.74	24.43	39.51	20.98	44.30	5.20	335.98	91.15
2013	23.04	21.02	37.53	21.11	44.64	5.69	240.75	103.14
2014	22.14	19.90	38.03	19.57	41.33	5.70	193.23	95.37
2015	23.38	18.88	38.45	18.65	40.06	3.16	215.50	106.74
2016	25.34	18.65	39.22	18.84	39.89	3.02	213.69	114.06
2017	25.72	16.82	39.80	19.13	37.01	3.10	171.47	73.66
2018	25.80	17.02	38.66	18.59	36.58	1.78	122.38	34.91
Total	25.41	21.94	39.08	19.87	41.60	5.65	250.02	124.68

Descriptive results indicate that the average level of vitamin D during the 2009–2018 years has been declining. While the average in 2009 was 41.00 ng/ml with the SD of 20.44 that has been decreased to 38.66 ng/ml with a SD of 18.59 in 2018. The total mean vitamin D level in these years in Iran is 39.08 ng/ml with a SD of 19.87 (Table 4).

The descriptive statistics involving mean and standard deviation for variables such as vitamin D level, age, and humidity, the sum of precipitation, in different provinces of Iran is displayed in Table 5. The findings show that the highest level of vitamin D is related to the West Azerbaijan with a mean of 33.24 ng/ml and a SD of 32.57 and the North Khorasan is recorded as the province with the lowest level of vitamin D with a mean of 14.46 ng/ml and a SD of 9.76 among 30 provinces of Iran. The level of vitamin D in other provinces was between 15.43 ng/ml and 30.31 ng/ml (Table 5). The mean age of population is 39.08 years. Ardabil has the lowest age with a mean of 17.61 years and a SD of 21.22 and Northern Khorasan has the highest age with a mean of 44.60 years and a SD of 32.14 (Table 5).

**Fig. 3:**
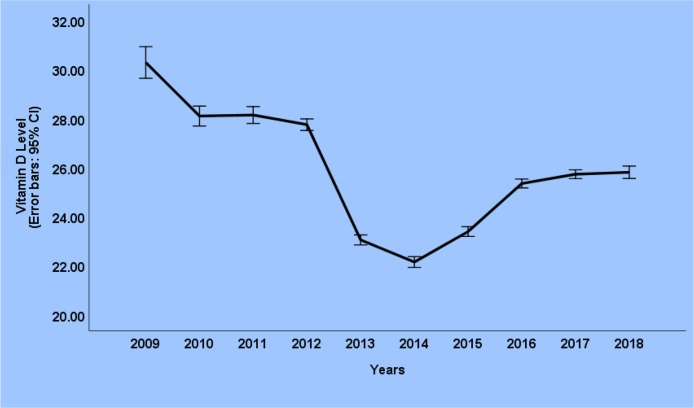
Trend of Vitamin D level in 10 years (2009–2018)

The trend of changes in vitamin D levels from 2009 to 2018 with a 95% confidence interval is shown in Chart 1. This figure indicates no dramatic changes from 2009 to 2012 (30.28 to 27.74), but in 2013 and 2014 there has been a rapid and vast decline in all over the country. From 2014 onwards, the average vitamin D trend has risen and by 2018, it remains about 25.80 ng / ml (Fig. 3).

**Table 5:** Descriptive statistics of vitamin D level, age, humidity, precipitation by different provinces of Iran

***Province***	***Vitamin D Level***		***Age (yr)***		***Humidity***		***Sum of precipitation***	
	***Mean***	***SD***	***Mean***	***SD***	***Mean***	***SD***	***Mean***	***SD***
Alborz	26.44	24.82	35.82	20.91	48.82	1.73	379.83	82.89
Ardabil	26.98	26.52	17.61	21.22	64.35	00.92	297.00	46.90
Chaharmahal and Bakhtiari	-	-	-	-	-	-	-	-
Bushehr	19.46	17.47	30.37	18.17	53.59	2.13	223.41	86.07
East Azerbaijan	25.65	20.30	34.15	20.29	53.45	2.46	273.42	49.70
Fars	30.31	32.78	32.50	20.45	33.58	2.41	252.83	52.39
Gilan	26.33	25.91	34.62	18.99	75.02	1.00	1013.02	103.79
Golestan	28.16	28.06	33.66	19.88	67.90	1.57	653.35	93.28
Hamadan	29.34	26.35	37.95	19.91	46.74	1.61	330.33	54.25
Hormozgan	20.19	19.82	33.72	19.32	54.82	3.30	139.10	61.10
Ilam	29.19	31.21	30.63	19.42	35.38	3.16	360.74	98.21
Isfahan	24.93	23.01	39.54	18.27	34.68	1.33	194.60	27.30
Kerman	22.13	22.33	42.04	19.08	31.84	1.42	162.56	35.86
Kermanshah	26.84	26.81	35.15	19.06	41.91	1.92	390.89	61.73
Khuzestan	22.30	23.62	36.76	19.42	40.72	1.94	285.40	66.47
Kohgiluyeh and Buyer-Ahmad	15.43	20.41	29.50	17.01	37.80	2.31	450.45	79.47
Kordestan	25.90	25.99	41.16	17.10	47.96	1.24	475.53	41.81
Lorestan	29.00	27.04	32.66	21.16	42.60	1.99	411.50	48.99
Markazi	26.18	23.73	37.50	19.99	40.07	1.11	249.15	25.28
Mazandaran	24.32	23.22	38.99	19.72	72.71	1.44	693.19	121.87
North Khorasan	14.46	9.76	44.60	32.14	51.35	00.00	210.74	00.00
Qazvin	27.75	27.11	36.32	19.88	50.96	2.16	317.91	68.86
Qom	23.50	18.01	37.88	17.87	37.14	00.00	101.58	00.00
Razavi Khorasan	23.12	23.12	37.29	18.47	43.67	1.97	200.56	31.27
Semnan	21.93	20.93	36.16	19.56	42.53	2.23	115.40	40.10
Sistan and Baluchestan	26.44	28.40	32.61	19.90	29.34	00.91	81.48	16.35
South Khorasan	28.52	27.77	30.06	18.65	31.86	2.34	104.95	15.25
Tehran	26.16	22.66	40.11	20.45	41.93	2.58	283.62	71.73
West Azerbaijan	33.24	32.57	39.33	18.87	52.58	1.80	395.83	16.54
Yazd	23.56	24.06	36.77	19.70	28.22	2.68	93.29	27.41
Zanjan	21.28	19.91	31.99	18.64	51.82	00.62	294.88	16.98
Total	25.41	21.94	39.08	19.87	41.60	5.65	250.02	124.68

**Fig. 4:**
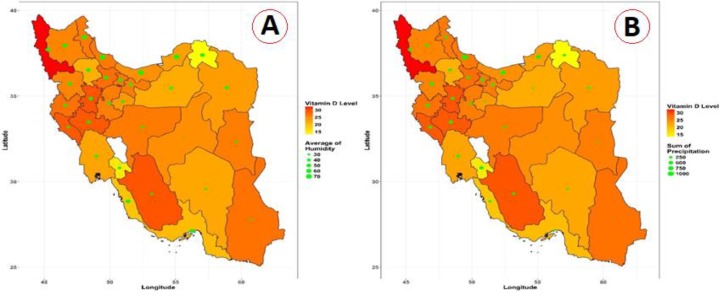
Panel A: Geographical distribution of the average level of vitamin D (Dark Red to yellow) and humidity (Green circles) Panel B: Geographical distribution of the average level of vitamin D (Dark red to yellow) and precipitation (Green circles)

The geographical distribution of vitamin D mean levels and the average of humidity and precipitation on Iran map from 2009 to 2018 is illustrated in Fig. 4. Vitamin D levels are shown in dark red to orange color. The high reddish is vitamin D level 100 and the less level of vitamin D is the brighter color. The green circles show the average humidity and the sum of precipitation of the province center. Bigger circles manifest that the humidity or sum of precipitation is higher and the small ones are associated with low humidity or sum of precipitation.

Pearson correlation coefficient was used to investigate the relationship between vitamin D level, age, gender, latitude and longitude, humidity and the sum of precipitation variables. The correlation between vitamin D and latitude was 0.027, which was significant (*P*<0.001). The correlation between vitamin D and longitude was 0.019, which means that by increasing of longitude, the vitamin D level increased too and it was significant (*P*<0.001). The correlation between humidity variable and vitamin D level was about −0.28. The negative sign means this was a reverse correlation, which expressed that increase of humidity, decreases the vitamin D level and it was also significant (*P*<0.001). With the increase in age, vitamin D levels have also increased.

There was a direct association between age and vitamin D, with a correlation coefficient of 0.223 which was statistically significant (*P*< 0.001).

The *t*-test was used to determine the difference in the mean level of vitamin D in men and women. It was 23.31 in the male group with a SD of 18.62 and 26.27 in women with a SD of 23.14. The difference between mean vitamin D level in men and women was statistically significant (*P* <0.001). Therefore, vitamin D levels in women were about 3 ng/ml higher than that of men.

## Discussion

The main goal of this descriptive study was to display information achieved from analysis of vitamin D levels of 30 different provinces of Iran. This article has the highest statistical population in terms of vitamin D research, so it can be a good reference for further investigations.

The global status of vitamin D was studied by Palacios et al. in a systemic review (26).

They investigated 103 published articles of ten years and based on it drew different maps of world vitamin d in various groups (Infants, children, adolescents, adults, pregnant or lactating woman, and elders). Table 6 demonstrates the finding of this study about Iran. It is also stated that vitamin D was especially low in women and girls in the Middle East (26).

**Table 6:** Iran Vitamin D status by Palacios et al. (26)

***Group***	***Vitamin D status***
Infants	93% (<30 ng/ml)(27)
Children	80% (<30 ng/ml)(28)
Adolescents	54% (<30 ng/ml)(29)
Adults	51% (<20 ng/ml)(30)
Pregnant and lactating women	67% (<20 ng/ml)(27)
Elders	80–85% (<30 ng/ml)(31)

Vitamin D level of 5232 people in 5 cities of Iran (Tehran, Tabriz, Mashhad, Shiraz and Bushehr) were examined (32). Accordingly, there was a deficiency in women and men of these cities, which was 75.1% and 72.1%, respectively. The severity of deficiency was higher in the elderly. Tehran had the highest deficit and Bushehr had the lowest (32). Their work shows the result for 5,232 people from just five cities of Iran, so we cannot generalize it to the whole country. We studied the number of 292,503 samples in 30 provinces of Iran and investigated the vast majority of the country.

Vitamin D level trend of 370 Iranian patients with the age of 35 and more were evaluated in a longitudinal study from 2001 to 2013 in three periods. Accordingly, the status of vitamin D had been improving toward 2013, but there was still a severe vitamin D deficiency (33). We studied all ages from newborns to the elders and did not follow their status in other years.

Tabrizi et al. reviewed 48 papers about vitamin D level of Iran from 2000 to 2016. In this systemic review and meta-analysis, vitamin D deficiency is defined as 61.90% in women, 45.64% in men and 60.45% in pregnant women. Moreover, they reported the diversity of vitamin D level in various geographical locations of Iran and the high prevalence of its deficiency all over the country (34). Our findings are similar to some parts that are founded in this article like the variety between Vitamin D levels in different geographical locations.

The number of women in the collected samples was about 2.5 times as high as men, which could be due to the improvement and promotion of women's healthcare, especially pre-pregnancy and lactation checkups. Since men with deficiency of vitamins have a higher incidence of 3 units more than women and this fact that fewer men are examined, paying more attention to men's health care and lifestyle is recommended (35).

As it can be deduced from the results of the study, the average total vitamin D level in Iran is 25.41 ng/ml, which is within the area of deficiency. Only 2 of the 30 surveyed provinces have normal vitamin D (6.6%) and the remaining 28 provinces are in the range of deficiency (93.4%). None of the provinces is within the severely deficient scale. Considering Iran is an Islamic country, the type of clothing is such that most of the body is covered, which causes the skin less exposed to sunlight (36, 37). Low level of ultraviolet radiation exposure is one of the main reasons for vitamin D deficiency (38). Variety in lifestyle and dietary habits (such as dairy and seafood consumption) also leads to variations in vitamin D level in different provinces.

A reduction in vitamin D level between 2012 and 2014 has been found on Fig. 3, which could be due to increased sanctions and, consequently, reduced drug entry and its raw material for production. Increase in vitamin D level from 2014 onwards could be related to the activities of the Ministry of Health and Medical Education in terms of improvement in health status and medicinal advances.

We had some limitations due to retrospective sampling. The samples were collected from laboratories and hospitals were not included, so the hospitalized patients who were severely ill are not involved. The referral cause of people to the laboratory was not clear so that we could not figure out how many people has disease and how many of them are healthy and they just wanted to do checkups. Also, since issues such as pregnancy, thyroid disease, recurrent abortion, MS, etc. are unknown in the samples, they cannot be isolated or examined.

## Conclusion

According to this study, Iran is facing vitamin D issues and most of the regions in Iran have a mean vitamin D level (m=25.41 ng/ml) in the deficiency range. Such results are very concerned, and it is recommended that the government provide special health plans to improve the status of vitamin D. Public awareness promotion, start of health care programs from schools, producing and consuming of vitamin D fortified foods, changing lifestyle and dietary habits, and adjusting the timing of exposure to sunlight as well as the use of complementary medicines under the supervision of a physician are some proposed approach to enhance the level of vitamin D levels.

It should be concerned that in addition to other associated factors, the genetic and epigenetic content of each person affects the amount of vitamin D. Hence, the study of these associations helps to a better understanding of one person's vitamin D status to determine the appropriate dose of supplementation required, which is somehow personalized medicine.

In the next studies, the correlation between the results of this article and average temperature in each province, ethnicity, elevation, food habits, and local jobs will be discussed in more details. More specific investigation on the reasons of vitamin D deficiency is needed to suggest approaches for improving the status of each province vitamin D status is a matter of urgency.

## Ethical considerations

This report is conducted with full respect for ethical issues and with the consent and permission of the laboratories.
